# Profiles of childhood maltreatment and peer victimization: associations with pathological internet use in Chinese left-behind children

**DOI:** 10.3389/fpsyt.2026.1767122

**Published:** 2026-02-16

**Authors:** Mingxin Li, Xin Dong, Wen Liu, Jiaqi Zhang, Hanbo Che, Changyuan Zhou

**Affiliations:** 1 College of Psychology, Liaoning Normal University, Dalian, China; 2 College of Education, Liaoning Normal University, Dalian, China

**Keywords:** childhood maltreatment, latent profile analysis, left-behind children, pathological internet use, peer victimization

## Abstract

**Introduction:**

Pathological Internet use is relatively common among adolescents, yet few studies have concurrently examined the influence of family-level and peer-related risk factors. This study aimed to use latent profile analysis to identify patterns of childhood maltreatment and peer victimization and to explore their associations with pathological Internet use.

**Method:**

In this cross-sectional study, 1,205 Chinese adolescents (*M* = 14.6, *SD* = 1.17; 20.1% left-behind children) completed self-report questionnaires. Participants completed validated instruments, including the Adolescent Pathological Internet Use Scale (APIUS), the Childhood Trauma Questionnaire-Short Form (CTQ-SF), and the Multidimensional Peer Victimization Scale (MPVS).

**Result:**

Latent profile analysis revealed three classes of childhood maltreatment and peer victimization: the low-risk group (80.83%), the severe peer victimization profile (11.86%), the severe childhood maltreatment with elevated peer victimization profile (7.31%). Compared with adolescents in the low-risk and severe peer victimization profiles, boys and left-behind children with migrant mothers were more likely to belong to the severe childhood maltreatment with elevated peer victimization profile. Moreover, adolescents in the severe childhood maltreatment with elevated peer victimization profile reported significantly higher negative outcomes of pathological Internet use than those in the low-risk profile.

**Conclusion:**

These findings underscore the value of jointly considering childhood maltreatment and peer victimization when identifying latent risk profiles, highlight the importance of differentiating specific left-behind family arrangements, and demonstrate the utility of examining distinct dimensions of pathological Internet use in capturing vulnerability to adverse outcomes.

## 1 Introduction

Since China’s Reform and Opening-up, a significant number of rural migrant workers have moved to economically prosperous cities in search of better employment opportunities. Due to their lower incomes and the various barriers they face in accessing public services—such as elementary education for their children, public medical care, and housing subsidies—many of these workers have to leave their children behind in their hometowns (1). This situation has led to the emergence of a new demographic group known as left-behind children (LBC). These children are defined as those under 18 years of age who remain in their hometowns while one or both of their parents migrate to urban areas for at least 6 months (2, 3).

Family and school, as the primary contexts of adolescent life, exert undeniable influences on their development (4). Within these settings, interpersonal stress emerges as a critical risk factor for psychopathology, manifesting through both distal pathways—such as childhood maltreatment within the family—and proximal pathways, such as peer victimization at school (5). Childhood maltreatment, encompassing physical abuse, emotional abuse, neglect, and negligent treatment by parents, guardians, or other household members, is associated with heightened externalizing behaviors, emotion dysregulation, depression, and anxiety among affected children (6). In addition, peer-related harm may also exert a negative impact on adolescents’ development. Peer victimization constitutes a manifestation of adverse peer relationships characterized by exposure to physical, verbal, property-related, or interpersonal aggression from peers (7). Extant literature emphasizes the significance of both physical and relational victimization, establishing that children experience peer maltreatment through both direct and indirect pathways (8). Therefore, this study examines peer victimization from both physical and relational perspectives.

Childhood maltreatment and peer victimization are associated with numerous adverse outcomes in adolescents, including internalizing problems (e.g., depression, suicidality), externalizing behaviors (e.g., aggression, substance abuse), and somatic conditions such as diabetes and heart disease (9–12). Research indicates that exposure to one form of victimization increases the risk of experiencing additional victimization types (13). Spillover theory suggests that children function within interconnected social systems (e.g., family and peer contexts), such that adversity in one domain can shape emotional and behavioral responses in others (14). Empirical evidence supports this view: a systematic review demonstrated bidirectional associations between child maltreatment and peer victimization (15), and longitudinal studies have shown that early physical or emotional abuse predicts later peer intimidation, physical assault, and social exclusion (16–18). Moreover, individuals exposed to any adversity subtype are likely to experience multiple forms of adversity (19). Collectively, these findings indicate a robust association between childhood maltreatment and peer victimization; however, research explicitly examining their co-occurrence remains limited (20).

However, despite robust evidence linking multiple forms of adversity, developmental outcomes following adversity exposure vary substantially across individuals. Children differ not only in their likelihood of experiencing adversity, but also in the specific types, combinations, and intensities of maltreatment and peer victimization they encounter. Whether distinguishing among these heterogeneous adversity profiles improves the precision of psychopathology risk estimation remains unclear. Latent profile analysis (LPA) is a person-centered analytic approach that identifies unobserved subgroups based on individuals’ patterns of responses to observed indicators (21). A key advantage of LPA is its capacity to capture heterogeneity in group membership by allowing variability both between and within profiles. Moreover, LPA enables predictors, covariates, and outcome variables to be incorporated into the model simultaneously, facilitating comprehensive examinations of profile correlates and consequences (22). Accordingly, LPA has been widely applied to investigate distinct configurations of childhood maltreatment and peer victimization. Using latent profile analysis, prior research has identified co-occurring profiles of family maltreatment and peer victimization ranging from low-risk to doubly disadvantaged patterns, with children exposed to both forms of adversity exhibiting the poorest psychosocial adjustment outcomes (23).The present study employs a person-centered approach to identify heterogeneous patterns of childhood adversity and to examine their implications for developmental outcomes.

Beyond identifying heterogeneous profiles of childhood maltreatment and peer victimization, it is also essential to consider how key sociodemographic factors shape individuals’ likelihood of belonging to different adversity profiles. Due to the absence of consistent parental supervision and family support, left-behind children occupy a particularly vulnerable developmental position and are at heightened risk of maltreatment by alternative caregivers, teachers, and community members (24, 25). Prolonged parental separation not only increases left-behind children’s exposure to caregiver maltreatment but also renders them especially susceptible to peer victimization (26). A meta-analysis further indicated that among different types of parental migration, children left behind by migrant mothers exhibited the highest levels of adverse developmental outcomes (27). In addition, substantial gender differences have been documented in peer victimization experiences, with girls more likely to experience emotional forms of peer abuse and boys more frequently subjected to physical peer abuse (28). Similarly, different forms of childhood maltreatment demonstrate distinct associations with depressive symptoms, and these associations vary by gender (29). Taken together, these findings underscore the importance of accounting for specific maltreatment types, gender, and left-behind status when examining patterns of childhood maltreatment and peer victimization, as doing so may yield a more nuanced and accurate characterization of risk across latent adversity profiles.

Beyond interpersonal adversity within family and peer contexts, contemporary adolescents also face emerging risks associated with the rapid expansion of digital environments. With the widespread adoption of Internet technology—particularly smartphones—the Internet has profoundly transformed and enriched daily life. According to the 54th Statistical Report by the China Internet Network Information Center (CNNIC), Internet penetration in rural China reached 69.2%, substantially lower than the 84.8% observed in urban areas, reflecting a persistent digital divide of 15.6 percentage points. During the same period, China added 7.42 million new Internet users, nearly half of whom were adolescents aged 10–19 years, accounting for 49% of all new users (30). Pathological Internet use (PIU) is commonly conceptualized as an impulse-control disorder characterized by heightened tension or arousal prior to Internet engagement and subsequent relief or gratification following use (e.g., online browsing), typically involving excessive and maladaptive Internet behaviors (31, 32). Adolescence represents a sensitive developmental period for the emergence of addictive behaviors (33). This vulnerability may be particularly pronounced among left-behind children, who experience prolonged parental separation and limited supervision and may rely on Internet use as a means of stress relief, coping with loneliness, or seeking social compensation (34, 35). Reduced parental supervision and heightened psychosocial stress should not be viewed as competing explanations; rather, diminished supervision may amplify adolescents’ reliance on the Internet as a coping strategy in response to loneliness, stress, and unmet emotional needs. Accumulating evidence indicates that pathological Internet use constitutes a significant risk factor for adolescents’ mental health, being associated with elevated depressive symptoms and an increased risk of suicidal ideation (36, 37).

According to the Social-Psychological-Physiological Model, vulnerability to Internet addiction arises from the dynamic interplay of social, psychological, and physiological factors (38, 39). Empirical research grounded in this framework has consistently shown that adverse experiences, particularly childhood maltreatment and peer victimization, substantially increase susceptibility to pathological Internet use. For example, Wei et al. (40) found that childhood maltreatment significantly predicted Internet addiction among college students, with this association moderated by self-control and gratitude. Similarly, Richard et al. (41) reported that childhood maltreatment was associated with heightened risk of problem gambling and disordered gaming behaviors. Peer victimization has also been identified as a salient predictor of pathological Internet use during adolescence. Chen et al. (42), for instance, demonstrated that peer victimization significantly predicted problematic online gaming among a large sample of adolescents, and Gini et al. (43) further showed that peer victimization directly contributes to adolescents’ involvement in cyberbullying behaviors. Taken together, these findings suggest that the effects of childhood maltreatment and peer victimization on pathological Internet use are closely intertwined rather than operating in isolation. Accordingly, the present study examines the joint associations of childhood maltreatment and peer victimization with pathological Internet use to provide a more comprehensive understanding of their combined influence on adolescents’ maladaptive Internet behaviors.

The current study aims to explore co-occurring patterns of different types of childhood maltreatment (e.g., physical abuse, emotional abuse, physical neglect, and emotional neglect) and peer victimization (e.g., physical and relational victimization) using latent profile analysis. Previous literature has highlighted the frequent co-occurrence of childhood maltreatment and peer victimization, emphasizing their compounded negative impact on children’s development (23). However, limited research has examined how these experiences coalesce in different groups, particularly in relation to the roles of gender and left-behind status. Specifically, this study seeks to identify distinct latent profiles of childhood maltreatment and peer victimization and to investigate how these patterns differ based on gender and left-behind status. Additionally, the study explores the relationship between these maltreatment and victimization profiles and adolescents’ pathological Internet use.

## 2 Method

### 2.1 Participants

The participants were 1,310 students from a junior high school. After excluding incomplete or invalid questionnaires (e.g., those with blank responses) (44), 1,205 valid questionnaires were obtained, yielding a validity rate of 91.98%. In this study, left-behind children were identified using the following questions: (1) “Do any family members migrate (leave the hometown) for work?” (2) “How often does your father return home?” (3) “How often does your mother return home?” Participants were classified as left-behind children if they were under 18 years of age and had at least one parent who had migrated to an urban area for work for six months or longer, while the child remained in their hometown. Based on parental migration status, left-behind children were further categorized as: (a) migrant father only (mother remained at home), (b) migrant mother only (father remained at home), and (c) both parents migrated. In our sample, 242 students (20.1%) were identified as LBC, including 164 with migrant fathers, 17 with migrant mothers, and 61 with both parents migrating. The remaining 963 students (79.9%) were non-left-behind children. The average age of the participants was 14.60 years (*SD* = 1.17). The sample included 597 boys (49.5%) and 608 girls (50.5%), with 397 students (32.9%) in the seventh grade, 497 (41.2%) in the eighth grade, and 311 (25.8%) in the ninth grade (as **Table 1**).

**Table 1:** Descriptive statistics of demographic variables (*N* = 1205).

Gender	Left behind status	Grade seven	Grade eight	Grade nine
Male	LBC	43	42	27
	NLBC	143	214	128
Female	LBC	47	57	26
	NLBC	164	184	130
Total		397	497	311

LBC, left-behind children; NLBC, Non-left-behind children.

### 2.2 Procedure

Data from junior high school students were collected in September 2024. A junior high school in Shandong Province was randomly selected, and twenty classes were subsequently randomly chosen from within this school. Data were collected during the class using a paper/pencil version survey administered to all students in this school classes. Research staff were trained before they administered the survey. The study was conducted in accordance with the Declaration of Helsinki as revised in 1989 and was approved by the Ethics Committee of Liaoning Normal University, the approval number is No. LL2024166. Student assent was obtained from both schools and parents.

### 2.3 Measures

#### 2.3.1 Pathological internet use

This study used the Adolescent Pathological Internet Use Scale (APIUS) developed by Li and Yang (31). The scale consists of 38 items and is divided into six dimensions: salience (e.g., Once I get online, I won’t think about anything else), mood alteration (e.g., When I encounter troubles, surfing the Internet can make me feel happier), social comfort (e.g., I feel safer communicating with others online), tolerance (e.g., In order to keep surfing the internet, I’d rather hold back my urine), compulsive internet use or withdrawal internet use (e.g., When I can’t get online, I really want to know what is happening online), negative outcomes (e.g., Surfing the Internet has had a negative impact on my health). The scale is scored on a five-point Likert scale. An average item score greater than or equal to 3.15 defines the PIU group, an average item score greater than or equal to 3 but less than 3.15 defines the PIU marginal group, and an average item score less than 3 defines the PIU normal group (5). This cutoff was proposed in the original scale development study based on empirical score distributions and symptom severity patterns in large adolescent samples and has been applied in prior studies of Chinese adolescents (45). Cronbach’s alpha coefficients were 0.767 (salience), 0.896 (mood alteration), 0.814 (social comfort), 0.820 (tolerance), 0.937 (compulsive or withdrawal-related Internet use), 0.814 (negative outcomes), and 0.959 for the total scale.

#### 2.3.2 Childhood maltreatment

This study used the Chinese version of Childhood trauma questionnaire (CTQ-SF) developed by Zhao et al. (46). The scale consists of 28 items to measure five dimensions: emotional abuse (e.g., At that time, some people at home called me “idiot”, “lazy” or “ugly”), physical abuse (e.g., At that time, someone at home hurt me badly and I had to go to the hospital), emotional neglect (e.g., At that time, someone in my family valued me), physical neglect (e.g., At that time, no one at home cared about my hunger) and sexual abuse (e.g., I was threatened to have sex with him/her at that time). Each item was rated on a five-point scale (1 = never, 2 = occasionally, 3 = sometimes, 4 = often, 5 = always). Items assessing emotional neglect and part of those assessing physical neglect were reverse-scored. Although the CTQ-SF has been widely used in Chinese samples, psychometric evidence suggests that the sexual abuse subscale may exhibit limited structural stability among Chinese adolescents, often necessitating item modification or removal to achieve acceptable validity (47). In addition, self-reported childhood sexual abuse in this population is characterized by low prevalence and cultural sensitivity. Accordingly, due to ethical considerations and potential measurement instability, the present study focused on four maltreatment dimensions—emotional abuse, physical abuse, emotional neglect, and physical neglect. In the current study, the Cronbach’s α were 0.682, 0.717, 0.793 and 0.662 for emotional abuse, physical abuse, emotional neglect and physical neglect respectively.

#### 2.3.3 Peer victimization

The Multidimensional Peer Victimization Scale (MPVS) developed by Mynard and Joseph (7) and translated and revised by Guo et al. (48) was used for measurement. Peer victimization was measured using two subscales from the multidimensional peer victimization scale. The physical victimization subscale (e.g., Some classmates threatened to hit me) and the relational victimization subscale (e.g., Some classmates provoke me to the relationship with other classmates, so that other classmates don’t like me) were rated on a 4-point scale, with response options ranging from 1 (never) to 4 (a lot). The Cronbach’s coefficients alpha in the current study were 0.822 and 0.900 for physical victimization and relational victimization, and 0.933 for the total scale.

### 2.4 Data analysis

Descriptive statistics and correlations for all study variables were calculated. Multicollinearity among the variables was considered by evaluating variance inflation factors (VIF). Latent profile analysis (LPA) was conducted using Mplus 8.3 to identify distinct profiles of adolescents based on their experiences of childhood maltreatment and peer victimization. Latent profile analysis is a person-centered approach designed to identify mutually exclusive, discrete latent profiles derived from a set of observed continuous indicators. In order to provide a much more intuitive interpretation, all study variables were converted into Z scores before conducting latent profile analysis. Models specifying one- through six-profile solutions were estimated and compared. After the optimal latent profile solution was identified, associations between demographic characteristics and profile membership were examined using the R3STEP approach (49). Specifically, a multinomial logistic regression was performed within the LPA framework, in which the latent profile variable was regressed simultaneously on gender and left-behind status. Finally, differences in pathological Internet use across the identified latent profiles were examined using the DU3STEP approach implemented in Mplus (50). Pathological Internet use and its specific dimensions were treated as continuous distal outcomes, while gender and left-behind status were included as covariates in the third step of the analysis. Overall profile differences were evaluated using omnibus chi-square tests, followed by pairwise comparisons conducted via MplusAutomation when significant effects were observed. This approach accounts for classification uncertainty in latent profile membership.

## 3 Results

### 3.1 Descriptive statistics and correlations

**Table 2** shows the descriptive and correlation of study variables. The variance inflation factor indicated that VIFs < 5 in the study, suggesting no significant multicollinearity issues (51). Childhood maltreatment is significantly positively correlated with peer victimization. All dimensions of childhood maltreatment and peer victimization are positively correlated with pathological Internet use.

**Table 2:** The correlation between variables (*N* = 1205).

Variables	1	2	3	4	5	6	7	8	9	10	11	12	13	14	15	16	17
1.Gender	1.00																
2. LBT	-0.04	1.00															
3.Age	-0.04	0.03	1.00														
4.Grade	-0.03	0.04	0.76^**^	1.00													
5.EA	0.05	-0.07^**^	-0.04	-0.05	1.00												
6.PA	-1.09^**^	-0.04	-0.02	-0.04	0.53**	1.00											
7.EN	-0.04	-0.01	0.01	0.02	0.24**	0.22**	1.00										
8.PN	-0.10^**^	-0.06^*^	0.06^*^	0.07^*^	0.28**	0.32**	0.55**	1.00									
9.PV	-0.18^**^	-0.08^**^	-0.01	0.04	0.35**	0.32**	0.12**	0.14**	1.00								
10.RV	0.002	-0.06	-0.02	-0.03	0.42**	0.29**	0.17**	0.20**	0.60**	1.00							
11.S	0.11^**^	0.09^**^	-0.02	-0.07^*^	0.25**	0.13**	0.10**	0.11**	0.30**	0.29**	1.00						
12.T	0.13^**^	0.12^**^	-0.01	-0.03	0.31**	0.22**	0.15**	0.19**	0.30**	0.33**	0.59**	1.00					
13.CIU	0.13^**^	0.10^**^	-0.03	-0.03	0.37**	0.23**	0.16**	0.20**	0.31^**^	0.38**	0.66**	0.81**	1.00				
14.MA	0.16^**^	0.09^**^	-0.01	-0.01	0.27**	0.09**	0.05	0.05	0.23**	0.27**	0.51**	0.48**	0.57**	1.00			
15.SC	0.13^**^	0.08^**^	-0.02	-0.02	0.28**	0.15**	0.10**	0.12**	0.25**	0.32**	0.48**	0.54**	0.61**	0.71**	1.00		
16.NO	0.10^**^	0.10^**^	-0.05	-0.07^**^	0.32**	0.24**	0.17**	0.23**	0.30**	0.36**	0.55**	0.78**	0.73**	0.36**	0.46**	1.00	
17.PIU	0.16^**^	0.12^**^	-0.03	-0.05	0.38**	0.22**	0.15**	0.19**	0.34**	0.41**	0.74**	0.85**	0.92**	0.75**	0.79^**^	0.79**	1.00
*M*	1.50	3.51	14.60	7.93	1.81	1.34	2.53	1.66	1.39	1.35	2.21	1.74	1.84	2.80	2.25	1.70	2.02
*SD*	0.50	1.05	1.17	0.76	1.01	0.76	1.32	0.78	0.56	0.49	0.98	0.80	0.87	1.13	0.96	0.68	0.72

^*^p < 0.05^**^ *p* < 0.01; Gender: 1 = boy, 2= girl; LBT, left-behind types, 1=left-behind children whose mother left; 2=left-behind children whose father left; 3=left-behind children whose both parents left. 4=non-left-behind children; Grade: 7, the seventh grade; 8, the eighth grade; 9, the ninth grade; EA, Emotional Abuse; PA, Physical Abuse; EN, Emotional Neglect; PN, Physical Neglect; PV, Physical Victimization; RV, Relational Victimization; S, salience; T, tolerance; CIU, compulsive internet use; MA, mood alteration; SC, social comfort; NO, negative outcomes; PIU, pathological Internet use.

### 3.2 Latent profile analysis model specification

**Table 3** presents the estimation of six latent profiles, ranging from a one-profile solution to a six-profile solution. The statistical analysis showed that the values of the Akaike Information Criterion (AIC), Bayesian Information Criterion (BIC), and adjusted Bayesian Information Criterion (aBIC) decreased as the number of specified profiles increased. For all profiles, entropy values above 0.8 indicated good profile separation, and the Bootstrap Likelihood Ratio Tests (BLRTs) were significant. Additionally, the AIC, BIC, and aBIC values consistently decreased with an increasing number of profiles (52).

**Table 3:** Fit indices for the various profile solutions.

Profiles	AIC	BIC	aBIC	Entropy	P(LMR)	P(BLRT)
1	20535.85	20596.98	20558.86			
2	18858.95	18955.74	18895.39	0.98	0.00	0.00
**3**	**18304.44**	**18436.89**	**18354.30**	**0.92**	**0.02**	**0.00**
4	17922.05	18090.16	17985.33	0.88	0.29	0.00
5	17548.14	17751.91	17624.85	0.90	0.35	0.00
6	17089.43	17328.86	17179.57	0.91	0.20	0.00

Bold type indicates final cluster solution. AIC, Akaike’s information criterion; BIC, Bayesian information criterion; A-BIC, sample-size-adjusted BIC; LMRT, Lo-Mendell-Rubin adjusted likelihood ratio test; BLRT, bootstrapped likelihood ratio test.

Although the four-profile model yielded slightly lower AIC and BIC values, the Lo–Mendell–Rubin test was no longer significant (*p* = 0.29), and entropy decreased from 0.92 to 0.88, indicating that the additional profile did not substantially improve model fit or interpretability. The three-profile model demonstrated a more parsimonious structure with clearer class separation and theoretical meaningfulness. Therefore, the three-profile model was retained as the optimal representation of the data.

**Figure 1:**
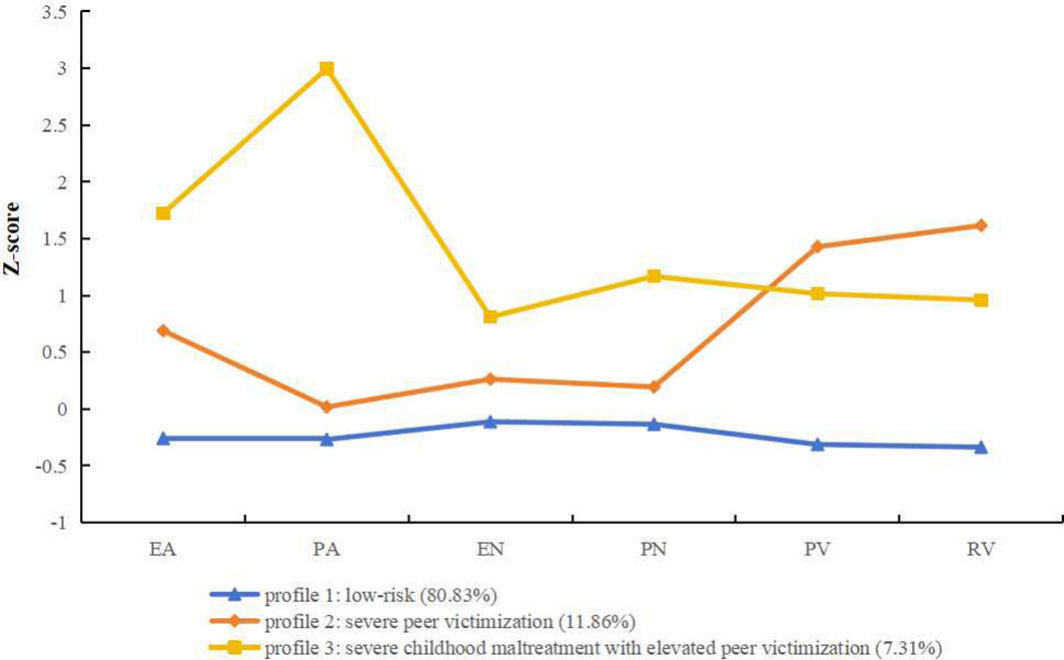
Mean Z-scores of childhood maltreatment and peer victimization for the three profiles solution. EA, Emotional Abuse; PA, Physical Abuse; EN, Emotional Neglect; PN, Physical Neglect; PV, Physical Victimization; RV, Relational Victimization.

**Figure 1** presents the levels of childhood maltreatment and peer victimization for the three profiles. The first profile reported low scores on both childhood maltreatment and peer victimization, and was therefore labeled as the “low-risk” profile (*n* = 974, 80.83%). The second profile showed relatively high scores on peer victimization and relatively low scores on childhood maltreatment, and was labeled the “severe peer victimization” profile (*n* = 143, 11.86%). The third profile demonstrated high scores on emotional and physical abuse, as well as neglect, with relatively high scores on peer victimization, and was labeled the “severe childhood maltreatment with elevated peer victimization” profile (*n* = 88, 7.31%).

### 3.3 Differences related to demographic characteristics

**Table 4** presents the distribution of gender and left-behind status across the three latent profiles of childhood maltreatment and peer victimization. Adolescents in the low-risk profile constituted the largest proportion of the sample. Notably, a substantial proportion of left-behind children were classified into this low-risk profile, whereas the severe peer victimization and the severe childhood maltreatment with elevated peer victimization profiles comprised comparatively smaller subgroups.

**Table 4:** Characteristics of the three profiles.

Variables	Low-risk (80.83%)	Severe peer victimization (11.86%)	Severe childhood maltreatment with elevated peer victimization (7.31%))
Gender			
Males	461	77	59
Females	513	66	29
Left-behind status			
left-behind children with migrant fathers	121	30	13
left-behind children with migrant mothers	10	2	5
left-behind children whose both parents left	40	17	4
non-left-behind children	803	94	66

To examine whether gender and left-behind status were associated with latent profile membership while accounting for classification uncertainty, a multinomial logistic regression was conducted using the R3STEP approach in Mplus. In this analysis, the retained three-profile latent variable was regressed simultaneously on gender (1 = male, 0 = female) and left-behind status (0 = non–left-behind children, 1 = left-behind children with migrant fathers, 2 = left-behind children with migrant mothers, 3 = left-behind children with both migrant parents). **Table 5** presents the regression estimates for gender and left-behind status predicting membership in the childhood maltreatment and peer victimization profiles. Significant differences were observed across profiles. Specifically, compared with adolescents in the low-risk and severe peer victimization profiles, boys were more likely to belong to the severe childhood maltreatment with elevated peer victimization profile. In addition, adolescents who were left behind by migrant mothers showed a higher likelihood of membership in the severe childhood maltreatment with elevated peer victimization profile than those who were not left behind.

**Table 5:** Multinomial logistic regression predicting latent profile membership by gender and left-behind status (R3STEP).

Variables	Low-risk vs severe peer victimization			Low-risk vs severe childhood maltreatment with elevated peer victimization			Severe peer victimization vs severe childhood maltreatment with elevated peer victimization		
	*B*(*SE*)	*t*	*OR*	*B*(*SE*)	*t*	*OR*	*B*(*SE*)	*t*	*OR*
Gender ^a^	0.23 (0.18)	1.28	1.25[0.80, 1.97]	**1.06 (0.29)**	**3.62**	2.88[1.38, 6.11]^***^	**0.83(0.33)**	**2.54**	2.30[0.99, 5.36]^**^
Left-behind status ^b^									
1	0.25(0.25)	1.03	1.29[0.68, 2.43]	0.33(0.37)	0.89	1.39[0.54, 3.57]	0.07(0.42)	0.17	1.08[0.37, 3.16]
2	0.25(0.80)	0.31	1.28[0.16, 9.96]	**2.04(0.61)**	**3.36**	7.66[1.61, 36.38]^***^	**1.79(0.91)**	**1.94**	5.99[0.58, 62.07]*
3	0.52(0.35)	1.50	1.68[0.69, 4.09]	-0.36(0.75)	-0.49	0.70[0.10, 4.76]	-0.88(0.80)	-1.11	0.41 [0.05, 3.32]

Results are from a multinomial logistic regression conducted using the R3STEP approach in Mplus, which accounts for classification uncertainty in latent profile membership. Values represent logit coefficients (B), standard errors (SE), t values, and odds ratios (OR) with 95% confidence intervals in brackets.

Gender was coded as 1 = male and 0 = female (reference group). Left-behind status was coded as 0 = non–left-behind children (reference group), 1 = left-behind children with migrant fathers, 2 = left-behind children with migrant mothers, and 3 = left-behind children with both migrant parents.

For each comparison, the first-listed profile serves as the reference category.

^*^p < 0.05; ^**^p < 0.01; ^***^p < 0.001. Bold values indicate statistically significant results.

### 3.4 Peer victimization and childhood maltreatment profile differences in pathological internet use

To examine whether pathological Internet use differed across latent profiles of childhood maltreatment and peer victimization, distal outcome analyses were conducted using the DU3STEP approach in Mplus. This procedure estimates differences in distal outcomes across latent profiles while accounting for classification uncertainty. All dimensions of pathological Internet use were treated as continuous distal outcomes, and gender and left-behind status were included as covariates in the third step of the model. After controlling for gender and left-behind status, a Wald χ^2^ omnibus test indicated a significant overall difference among the three profiles in the negative consequences dimension of pathological Internet use, χ^2^ (2) = 6.05, *p* = 0.04 (see **Table 6**). Subsequent pairwise comparisons showed that adolescents in the severe childhood maltreatment with elevated peer victimization profile reported significantly higher levels of negative consequences than those in the low-risk profile. Differences between the severe peer victimization profile and the other two profiles were not statistically significant. For the remaining dimensions of pathological Internet use—including salience, tolerance, compulsive Internet use, mood alteration, social comfort, and overall pathological Internet use—the omnibus Wald χ^2^ tests were not significant. Consistent with these results, pairwise comparisons revealed no significant differences between any of the latent profiles on these dimensions after controlling for gender and left-behind status.

**Table 6:** Latent profiles differences in pathological internet use dimensions (DU3STEP).

Variables	Profiles of peer victimization and childhood maltreatment						
	Low-risk		Severe peer victimization		Severe childhood maltreatment with elevated peer victimization		
	*M*	*SE*	*M*	*SE*	*M*	*SE*	χ^2^
salience	2.19	0.03	2.26	0.08	2.32	0.13	1.35
tolerance	1.63	0.06	1.74	0.03	1.87	0.10	4.48
compulsive internet use	1.83	0.03	1.82	0.07	1.95	0.11	1.34
mood alteration	2.80	0.04	2.82	0.09	2.82	0.14	0.08
social comfort	2.24	0.03	2.30	0.08	2.35	0.12	1.30
negative outcomes	1.67	0.02	1.77	0.06	1.86	0.09	6.05^*^
pathological Internet use	2.07	0.02	2.13	0.06	2.21	0.10	2.10

^*^p < 0.05.

## 4 Discussion

Using latent profile analysis, this study identified three distinct patterns of childhood maltreatment and peer victimization among junior high school students: a low-risk profile, a severe peer victimization profile, and a severe childhood maltreatment with elevated peer victimization profile. The findings further revealed meaningful gender and left-behind status differences in profile membership, with boys and left-behind children with migrant mothers being more likely to belong to the severe childhood maltreatment with elevated peer victimization profile. Notably, adolescents in this profile reported substantially higher levels of negative outcomes of pathological Internet use, underscoring the heightened vulnerability associated with cumulative and severe interpersonal adversity.

### 4.1 Latent profiles of childhood maltreatment and peer victimization

This experimental investigation identified three distinct co-occurrence patterns of childhood maltreatment and peer victimization through latent profile analysis: low-risk profile (80.83%), severe peer victimization profile (11.86%), and severe childhood maltreatment with elevated peer victimization profile (7.31%). Although the number of profiles differs from previous studies—such as the longitudinal UK–US investigation identifying four groups (15) and the six-profile solution reported by Fu et al. (23) among Chinese adolescents—these discrepancies likely reflect regional, developmental, and methodological variations. Importantly, our findings converge with prior research in demonstrating that childhood maltreatment and peer victimization frequently co-occur (23, 53). It should be noted that the profile characterized by severe childhood maltreatment and elevated peer victimization represents a relatively small proportion of the overall sample (7.31%). Rather than indicating population-level prevalence, this subgroup reflects a high-risk, high-need minority of children who are simultaneously exposed to adversity within both family and peer contexts. The identification of this subgroup provides empirical support for spillover theory, which posits that dysfunction in one social system (e.g., the family) may heighten vulnerability in other contexts (e.g., peer relationships). Children experiencing severe maltreatment may develop emotional dysregulation and maladaptive interpersonal behaviors that increase their susceptibility to peer victimization, thereby accumulating risks across contexts (54, 55). Consistent with prior literature, the majority of children in the present sample were classified into the low-risk profile, experiencing little to no maltreatment or peer victimization (23). This pattern aligns with evidence suggesting that most children are not chronically exposed to victimization, while a relatively small subset experiences multiple, overlapping forms of adversity. From a practical perspective, these findings underscore the importance of moving beyond population-level averages to identify and support children within this particularly vulnerable subgroup. Targeted screening and intervention efforts focusing on children exposed to both family maltreatment and peer victimization may be especially critical for preventing long-term maladjustment.

### 4.2 Demographic characteristics of childhood maltreatment and peer victimization

Gender and the type of left-behind status can predict profiles of childhood maltreatment and peer victimization. Boys were more likely to belong to the severe childhood maltreatment with elevated peer victimization profile compared to girls, suggesting a heightened exposure to multiple forms of adversity. This finding is consistent with prior Chinese studies showing that boys tend to experience more frequent and diverse forms of victimization (56). This gender difference may be explained by traditional childcare patterns in China, where families often have higher expectations for boys. Parents expect boys to succeed and carry on the family name, becoming primary providers for their families and aging parents in adulthood (55). Moreover, previous research has consistently shown that boys are more vulnerable to peer victimization than girls (57). This gender disparity may be attributable to differences in peer interaction patterns. Compared with girls, boys typically engage in peer groups that are larger, more loosely structured, and more competitive, which increases both the frequency and intensity of aggressive interactions among peers (58). In contrast, prevailing social norms and moral expectations often position girls as a protected group, affording them greater adult supervision and social buffering, which may reduce their risk of peer victimization (59).

The present study further revealed that approximately 70% of left-behind children were classified into the low-risk group. However, compared with non-left-behind children, adolescents whose mothers had migrated for work were significantly more likely to be assigned to the dual-risk profile characterized by severe childhood maltreatment and elevated peer victimization. This finding underscores the particularly salient role of maternal absence in shaping children’s exposure to cumulative adversity across family and peer contexts. In the Chinese cultural context, mothers are typically the primary caregivers and are more directly involved in children’s daily supervision, emotional support, and socialization (60). Maternal migration may therefore disrupt consistent caregiving, reduce emotional availability, and weaken monitoring of children’s peer interactions, thereby increasing vulnerability to both maltreatment within the family and victimization in peer settings (27). Compared with mothers who migrate to distant urban labor markets and thus are absent from daily caregiving, fathers who remain behind often have fewer caregiving resources, weaker emotional engagement, and lower motivation or capacity to fulfill the traditional breadwinner-caretaker role, which may exacerbate developmental disadvantages among left-behind children (61). From a family systems perspective, maternal absence may also exacerbate stress within the caregiving environment, particularly when alternative caregivers such as fathers or grandparents face limited resources, caregiving capacity, or emotional closeness with the child. These strains may heighten the risk of harsh, neglectful, or inconsistent parenting practices, increasing the likelihood of childhood maltreatment. Concurrently, insufficient parental guidance and emotional support may impair children’s emotion regulation and social competence, which are critical protective factors in peer relationships, thereby elevating the risk of peer victimization. The co-occurrence of family maltreatment and peer victimization among children with migrant mothers thus provides further support for a spillover process, whereby adversity in the family context increases susceptibility to harm in peer environments (62).

Taken together, the findings of the present study indicate that both gender and differentiated forms of left-behind status are associated with distinct patterns of exposure to childhood maltreatment and peer victimization. Rather than exerting uniform effects, these sociodemographic factors appear to shape children’s vulnerability in nuanced ways, with certain subgroups—such as boys and children experiencing maternal absence—showing elevated risk for compounded adversity across family and peer contexts. Prevention and intervention efforts may benefit from tailored strategies that account for heterogeneity in children’s developmental contexts, enabling more precise identification of high-need subgroups and more effective allocation of support and resources.

### 4.3 Pathological internet use differences across profiles of childhood maltreatment and peer victimization

Consistent with prior research, adolescents exposed to childhood maltreatment and peer victimization are at elevated risk for problematic patterns of Internet use (42, 63). However, the present findings extend this literature by demonstrating that, after controlling for gender and left-behind status, differences across latent profiles emerged only in the negative consequences dimension of pathological Internet use. Specifically, adolescents characterized by co-occurring high levels of childhood maltreatment and peer victimization exhibited significantly greater functional impairments related to Internet use than those in the low-risk profile. This pattern suggests that cumulative interpersonal adversity may not necessarily translate into higher levels of compulsive or emotionally driven Internet use, but rather manifests in more pronounced disruptions to daily functioning, such as academic difficulties, interpersonal conflicts, and reduced engagement in offline activities (64). Importantly, this pattern suggests that adolescents exposed to cumulative family and peer adversity may not primarily engage in Internet use for heightened pleasure or social comfort, but instead experience greater difficulty containing Internet use within adaptive boundaries. As a result, negative outcomes may represent a more proximal and sensitive manifestation of risk than motivational or affective PIU components in this population. Adolescents who have experienced peer victimization and childhood maltreatment are more likely to develop intense negative emotions and may initially use the Internet as an accessible coping resource to regulate these feelings. However, prolonged reliance on this strategy can undermine real-life emotional regulation and daily functioning, leading to observable negative consequences (65). In addition, the co-occurrence of family- and peer-level victimization may compromise adolescents’ self-esteem and social competence, limiting their capacity to regulate Internet use effectively and increasing their vulnerability to maladaptive outcomes (66). While prior studies have focused on global Internet addiction outcomes, our finding emphasizes the need to consider specific functional impairments as a sensitive indicator of risk in adolescents exposed to complex adversity profiles.

### 4.4 Research implications and limitations

This study employed latent profile analysis to concurrently examine childhood maltreatment and peer victimization, identifying distinct risk profiles based on these adverse experiences. From a practical perspective, intervention strategies can be tailored to each latent profile. For instance, adolescents in the severe peer victimization profile may benefit from peer support programs and social skills training, whereas those in the severe childhood maltreatment with elevated peer victimization profile may require combined family interventions, emotion regulation training, and targeted psychosocial support to mitigate the compounded risks. For left-behind children, particularly those experiencing maternal absence, interventions may need to further emphasize enhancing caregiving quality, emotional support, and monitoring within the home to buffer both family- and peer-related adversities. However, this study still has some limitations. First, childhood maltreatment, peer victimization, and pathological Internet use are dynamic experiences that may change across developmental periods. The cross-sectional design precludes the examination of temporal relationships and causal inferences, and may also be subject to quantitative biases such as classification uncertainty inherent in single-wave latent profile analyses. Future research could address these limitations by implementing multi-wave longitudinal designs using trajectory-based or transition-based mixture models, which allow for examination of changes in risk profiles over time, the stability of latent classes, and the effects of time-varying covariates while reducing potential bias. Second, the reliance on self-report measures may introduce common method bias and social desirability effects. Future research could incorporate multiple informants or behavioral indicators to enhance measurement validity. Third, the sample was drawn from middle school students in a specific region of China, which may limit the generalizability of the findings; replication across diverse regions and cultural contexts is recommended. Lastly, this study focused solely on two types of adverse experiences and did not consider other important factors—such as family functioning, emotion regulation, or school context—that may interact with adversity to influence Internet use. Future studies should integrate broader ecological variables to develop a more comprehensive risk framework.

## 5 Conclusion

The present findings suggest that childhood maltreatment and peer victimization may co-occur in distinct patterns rather than uniformly across adolescents. Boys and left-behind children with migrant mothers were particularly likely to belong to the severe childhood maltreatment with elevated peer victimization profile, which was associated with elevated negative outcomes of pathological Internet use across multiple dimensions. These results underscore the importance of integrative person-centered approaches, finer distinctions in left-behind family arrangements, and multidimensional assessments of pathological Internet use in capturing adolescents’ vulnerability to cumulative interpersonal adversity.

## Data Availability

The raw data supporting the conclusions of this article will be made available by the authors, without undue reservation.
